# Supplementary material for: A Likelihood Approach for Real-Time Calibration of Stochastic Compartmental Epidemic Models
Source: PLoS Comput Biol. 2017 Jan 17;13(1):e1005257. doi: 10.1371/journal.pcbi.1005257 (PMC5240920; doi:10.1371/journal.pcbi.1005257)
Supplement: S1 File — (TAR.GZ) [file pcbi.1005257.s014.tar.gz › HSPH_Online-SI-Revision/Additions/MSS11/n100-extreme-SEITR_noisy/n100-extreme-SEITR_noisy_NEW2_summary2.pdf]

peak -8	3w spec	3w cum	5w spec	5w cum	end	3w spec LOG	3w cum LOG	5w spec LOG	5w cum LOG	end LOG
mean	402.9	461.5	100.2	136.1	39.3	1.6	1.4	2.1	1.8	1.1
median	100.	97.8	95.7	97.2	40.9	1.8	1.5	2.3	2.	1.1
quantile .1	63.5	61.9	58.1	60.2	22.6	0.7	0.7	0.8	0.7	0.3
quantile .9	1407.8	1673.4	130.8	264.1	47.3	2.	1.8	2.4	0.7	1.7
20	20	15	18	4						

peak -4	3w spec	3w cum	5w spec	5w cum	end	3w spec LOG	3w cum LOG	5w spec LOG	5w cum LOG	end LOG
mean	20.9	20.	19.9	17.	15.2	0.1	0.1	0.1	0.1	0.1
median	16.	14.7	17.3	14.4	13.7	0.1	0.1	0.1	0.1	0.1
quantile .1	5.4	3.4	8.9	6.1	5.7	0.	0.	0.	0.	0.
quantile .9	37.8	34.3	32.6	31.5	25.1	0.2	0.2	0.2	0.	0.1
15	17	3	12	3						

peak	3w spec	3w cum	5w spec	5w cum	end	3w spec LOG	3w cum LOG	5w spec LOG	5w cum LOG	end LOG
mean	17.	15.2	26.	15.5	16.1	0.1	0.1	0.1	0.1	0.1
median	12.7	14.4	17.7	13.3	13.1	0.1	0.1	0.1	0.1	0.1
quantile .1	2.6	6.3	5.	4.8	3.1	0.	0.	0.	0.	0.
quantile .9	32.6	25.2	53.5	27.	30.5	0.2	0.1	0.3	0.	0.2
5	1	4	1	1						

peak +4	3w spec	3w cum	5w spec	5w cum	end	3w spec LOG	3w cum LOG	5w spec LOG	5w cum LOG	end LOG
mean	37.8	24.5	69.2	28.6	32.7	0.1	0.1	0.2	0.1	0.1
median	24.2	17.9	35.2	20.7	24.4	0.1	0.1	0.2	0.1	0.1
quantile .1	5.9	3.	7.7	2.5	5.2	0.	0.	0.	0.	0.
quantile .9	67.5	53.1	106.4	58.2	60.2	0.3	0.2	0.4	0.	0.3
6	3	6	1	2						

peak +8	3w spec	3w cum	5w spec	5w cum	end	3w spec LOG	3w cum LOG	5w spec LOG	5w cum LOG	end LOG
mean	159.3	77.9	377.6	85.3	84.4	0.3	0.2	0.4	0.2	0.2
median	46.	38.6	70.	40.	41.8	0.2	0.2	0.3	0.2	0.2
quantile .1	11.2	6.2	15.3	8.1	6.3	0.1	0.	0.1	0.	0.
quantile .9	195.7	132.6	483.6	147.2	153.7	0.5	0.4	0.8	0.	0.4
6	3	9	3	3	{}	{}	{}	{}	{}	{}
